# How does decision-making change during challenging times?

**DOI:** 10.1371/journal.pone.0270117

**Published:** 2022-07-29

**Authors:** Alessandro Cicerale, Enrico Blanzieri, Katiuscia Sacco

**Affiliations:** 1 BraIn Plasticity and Behavior Changes @Department of Psychology, and Neuroscience Institute of Turin—University of Turin, Turin, Italy; 2 Department of Engineering & Information Sciences, University of Trento, Trento, Italy; Dartmouth College, UNITED STATES

## Abstract

Prospect Theory, proposed and developed by Kahneman and Tversky, demonstrated that people do not make rational decisions based on expected utility, but are instead biased by specific cognitive tendencies leading to neglect, under- or over- consider information, depending on the context of presentation. In this vein, the present paper focuses on whether and how individual decision-making attitudes are prone to change in the presence of globally challenging events. We ran three partial replications of the Kahneman and Tversky (1979) paper, focusing on a set of eight prospects, after a terror attack (Paris, November 2015, 134 subjects) and during the Covid-19 pandemic, both during the first lockdown in Italy (Spring 2020, 176 subjects) and after the first reopening (140 subjects). The results confirm patterns of choice characterizing uncertain times, as shown by previous literature. In particular, we note significant increase of risk aversion, both in the gain and in the loss domains, that consistently emerged in the three replications. Given the nature of our sample, and the heterogeneity between the three periods investigated, we suggest that the phenomenon we present can be explained stress-related effects on decision making rather than by other economic effects, such as the income effect.

## Introduction

Decision-making under uncertainty is an important topic of research with implications that span well beyond the domain of academic psychology: it has obvious consequences for finance [1], economics [2], policy-making [3] and many other domains where individual choices play a central role.

For much of the 20th century, expected utility theory (EUT) has been the mainstream approach to the study of decisions in risky or uncertain contexts and its original formulation posits that individuals make their choices based on the comparison between expected utility values [4]. One of the most successful descriptions of human decision-making under uncertainty, however, has been prospect theory (PT) [5], together with its refinement, the Cumulative Prospect Theory (CPT) [6]. Tversky and Kahneman analyzed the answers of a sample of participants to a set of financial decision-making problems, inspired by paradoxes in the choice patterns initially discovered by Allais [7]. They documented a set of deviations from the predictions of EUT, such as utility functions depending on the domain of reference (concave in the gain domain and convex in the loss domain). Besides, they showed specific ‘paradoxes’, such as the preference for small certain gains over large uncertain gains even when the expected outcome is identical (certainty effect); the tendency to be risk seeking when trying to maximize gains, but risk averse when trying to minimize losses (reflection effect); the shifting in preference patterns when the same outcome is presented in terms of gains rather than losses from a reference point (framing effect).

These results have drawn enormous interest from both psychologists and economists but, up until recently, systematic large-sample replications of the original studies were missing. Recently, a large replication study by Ruggeri and colleagues [8] substantially confirmed the original findings, albeit with attenuated effects, in a study enrolling 4098 participants from 19 countries. While this recent publication confirms the descriptive power of CPT in a set of circumstances, a series of works noted that decision-making can be affected by various factors, including events that have an impact at the collective level. For instance, Sacco and colleagues [9] studied decision patterns in the aftermath of the 9/11 terrorist attacks. While the authors confirmed the majority of the results originally reported by Kahneman and Tversky, they also found evidence of more widespread risk aversion, both in the gain and in the loss domains, as well as the loss of the reflection and framing effects for some pairs of prospects. In line with these results, a study [10] showed that the heightened fear of dread risks (low-probability, high-consequence events) linked to air travel after 9/11 led to an increased preference for land transportation (and, paradoxically, driving accidents). However, other experiments [11] carried out after heavy snowfalls and an earthquake in China (Wenchuan earthquake, 2008) did not completely replicate these tendencies: while finding increased risk-aversion in the loss domain, they also found an increased preference for small probabilities in the gain domain. Recently, one study [12] explored the interaction between risk attitudes during the COVID-19 pandemic and previous life experiences, suggesting that the impact of the pandemic becomes influential only in those participants who had been previously affected by negative life events.

The goal of this paper is by no means an attempt to verify or falsify PT; rather, it focuses on the short- and medium-term effects of external shocks on choice patterns. Indeed, although individual risk preferences are assumed to be stable over time by classical economic theories, more recent experimental work showed that risk preference can vary with time [13]. In this study, we intended to replicate what Sacco and colleagues found following the September 2001 terror attacks, as they can be seen as a prototype of globally challenging events with a potential impact on risk preferences. Indeed, similar cognitive effects could occur following other disruptive events. In this paper we investigate risk preferences using a set of economic prospects, both in the gain and loss domain. In particular, we focused on the prospects where Sacco and colleagues found significant differences from the patterns identified by Kahneman and Tversky [5]. The differences between the results of the two works are briefly summarized here. When probabilities of medium entities of winning or losing money after an initial gain are compared with sure options (Prospects A and A’ in the present paper), the preference for a sure win of minor entity was still present, while the preference for a probable loss of major entity was not replicated, suggesting heightened loss aversion. When dealing with very unlikely events vs. sure small gain/loss (Prospects B and B’) the overweighting of suffering a large loss with very low probabilities (the tendency leading to pay high insurance premiums) was still present, while the overweighting of a large gain with very low probabilities (tendency leading to buy lottery tickets) was not replicated–in line with national lottery ticket sales. When the two options involve small, quite similar options (Prospects C and C’), the preference for the slightly lower probability of winning a bigger amount of money disappeared, while the tendency to prefer the slightly higher probability of losing a smaller amount of money was similar. When the two options involve very unlikely events (Prospects D and D’) both the preference for the less probable event of winning a bigger amount of money and the more probable event of losing a smaller amount of money were not replicated; on the contrary, an opposite tendency of preferring most probable events emerged in the gain domain. Such deviations from PT predictions were interpreted by the authors as the effect of the catastrophic event and the subsequent change in decision-making attitudes: “a shared loss biases decision-making in favor of a search for security” [9].

To increase the generalizability of the results, we recorded data during two separate events: the Paris terror attack in 2015 (Experiment 1) and the COVID-19 pandemic (Experiment 2a: data collected during the first lockdown in Spring 2020; and Experiment 2b: data collected during the subsequent reopening phase in Summer 2020).

Furthermore, we explored some other possibly impacting factors: personality traits and severity of the crisis. The reason to explore possible correlations between personality traits and decision-making derives from the presence of contrasting results in the relative literature. Some papers found that personality traits significantly correlate with risk-taking [14–16] and that, in the financial domain, extraversion is linked with a higher likelihood to pay excessive prices for risky assets in a simulated market while high neuroticism is linked to holding fewer risky assets [17]. Other studies, however, did not confirm such results [18]. It is therefore worth investigating whether personality traits have or do not have an impact on decision-making processes during periods of crisis characterized by increased uncertainty. To check whether the severity of the pandemic had an impact on risk preferences, we used the daily increase of deaths, active cases of COVID-19 and Emergency Room (ER) admissions, as well as self-reported fear about the pandemic as measures of severity.

Finally, this work also affords us the opportunity of answering an issue raised by Li and colleagues [11] who pointed out that, by converting US dollars into Italian Liras, Sacco et al. modified the magnitude of the values included in the prospects (5$ became 30000£), possibly introducing a confounding factor. As euros and dollars are expressed in with amounts of the same order of magnitude, this paper can shed light on this issue.

## Materials and methods

The 4 pairs of prospects used in the present study (see Table 1 for a list) are those where Sacco and colleagues [9] found a distribution of answers different from that predicted by the CPT, thus representing decision-making attitudes during periods of global uncertainty. The questions are reported below and were used in both Experiment 1 [Bataclan] and Experiment 2 [Covid]. The monetary values were based on the ones previously used, adjusted for inflation to 2020 values using the official tool provided by the Italian Bureau of Statistics (Istituto Nazionale di Statistica–tool available at http://rivaluta.istat.it), converted to euros and rounded to the most significant digit (e.g.: 3000€ instead of 3120€).

**Table 1:** Pairs of prospects administered in the present study.

Pair	Prospect	Question
1	A	You have been given 4000€. Now choose between a 50% possibility of winning 4000€ or a sure win of 2000€
	A’	You have been given 8000€. Now choose between a 50% chance of losing 4000€ or a sure loss of 2000€
2	B	Choose between one possibility out of 1000 of winning 30000€ or a sure winning of 30€
	B’	Choose between one possibility out of 1000 of losing 30000€ or a sure loss of 30€
3	C	Choose between a 20% possibility of winning 4000€ or a 25% chance of winning 3000€
	C’	Choose between a 20% possibility of losing 4000€ or a 25% chance of losing 3000€
4	D	Choose between one possibility out of 1000 of winning 6000€ or two possibilities out of 1000 of winning 3000€
	D’	Choose between one possibility out of 1000 of losing 6000€ or two possibilities out of 1000 of losing 3000€

For Experiment 2 only, we also administered an Italian version of the 10-item Big Five personality Inventory [19]. As well as this, we collected data related to the ongoing COVID-19 pandemic: the numbers of patients in intensive care, variation in the number of COVID positives and number of deaths were obtained through an official open-source repository, maintained by the emergency management agency of the Italian Government (Dipartimento della Protezione Civile). Furthermore, participants were asked two questions about their worry levels about the pandemic (‘How much are you worried about the economic consequences of the COVID-19 pandemic?’, and ‘How much are you worried about the health consequences of the COVID-19 pandemic?’), as well as one question about their level of knowledge (How much are you informed about the COVID-19 pandemic?). The questions were rated using a 5-points Likert scale. Table 2 reports descriptive statistics for the personality traits and the pandemic-related questions.

**Table 2:** Descriptive statistics–personality factors and pandemic-related questions.

	N	Min	Max	mean	Std. Dev.
Agreeableness	316	2	10	6.02	1.73
Consciousness	316	2	10	7.18	1.70
Stability	316	2	10	5.36	1.94
Extraversion	316	2	10	5.77	1.78
Openness	316	2	10	7.44	1.79
Worry for economic consequences	313	1	5	3.98	0.96
Worry for health consequences	313	1	5	3.62	0.97
Knowledge self-rating	313	1	5	3.81	0.78

### Participants and procedures

#### Experiment 1–2015 Paris terror attacks (Bataclan)

The experimental sample consisted of students from the Faculty of Psychology, Università di Torino (Turin, Italy). Participants received a paper form and answered the questions by marking their preferred options. The answers were then manually transcribed on an electronic spreadsheet, checked and stored. We created eight sequences (four were randomly generated orders, four were obtained through reversing the random orders), and participants randomly received one of the eight possible forms. We received 134 fully filled out forms (mean age 19.8 years, SD 1.6; 75% women), transcribed the answers electronically and stored the results for further elaborations.

#### Experiment 2 –Covid-19

A list of 922 university students was drafted in March 2020. The list included students enrolled at the Pontificia Università Salesiana–IUSTO Rebaudengo and at the Università degli Studi di Torino (Turin, Italy). The students were pooled together. Students were randomly assigned to one of four groups, receiving the first invitation respectively on March 23rd, April 26th; July 26th and September 21st. We received 92 and 84 answers from the first two groups (mean age 22.4 years, SD 4.7, 85% women); 65 and 75 answers from the last two groups (mean age 22.2 years, SD 3.6, 78% women). Participants received an invitation via e-mail, with a brief explanation of the study. The experiment was entirely conducted online due to COVID-19 limitations, and all questions were administered through a LimeSurvey-hosted questionnaire. The questions were administered in a fully randomized order, and the answer options were randomized.

To verify whether our results differed from the recent replication of Kahneman and Tversky’s results [8], we computed expected proportions of answers from two age-matched subsamples from their publicly available dataset. The first one only included 117 Italian subjects aged 18–25 years old (mean age = 22.11, 52% men), while the second one expanded the sample to include all countries (N = 1425, mean age = 22.47, 48% men). The same approach was used to compare our results with the study of risk preferences after the 9/11 attacks [9]. The study was approved by the internal review board of the Università degli Studi di Torino—Prot. n. 142238. In order to take part in the study participants had to explicitly confirm that they had read the informed consent and accepted participating in the study.

### Data analysis

The data were analysed using SPSS 26.0. We compared the answers to each question using a chi-squared test against a uniform distribution. For Experiment 2, we also carried out point-biserial correlations between the answer to the questions and personality scales, as well between the answers and the COVID-19 data. Chi squared tests were also used to compare our samples with the frequencies expected based on [8].The results of the chi-squared were corrected for multiple comparisons, experiment-wise, using the Holm-Bonferroni method [20]; the results of the correlations were corrected using the False Discovery Rate approach [21].

## Results

### Experiment 1: Decision-making after the 2015 Paris terror attacks

#### Response distributions

The response distribution differed substantially from chance for items A (χ^2^ = 36.56, p_c_<0.001), B’ (χ^2^ = 36.56, p_c_<0.001), but not for A’ (χ^2^ = 2.99), B (χ^2^ = 1.91), C(χ^2^ = 0.74), C’ (χ^2^ = 5.05) D (χ^2^ = 0.27) or and D’ (χ^2^ = 3.61, all corrected p values non-significant). There was no association between gender and the answers to any prospect (all p_c_ > 0.05). 76% of the respondents chose the option with the highest probability of happening in prospect A, 57% in prospect A’, 56% in prospect B, 76% in prospect B’, 46% in prospect C, 60% in prospect C’, 48% in prospect D and 42% in prospect D’.

### Experiment 2: Decision-making during the COVID-19 pandemic

#### Response distributions (1^st^ time period—lockdowns)

**Fig 1:**
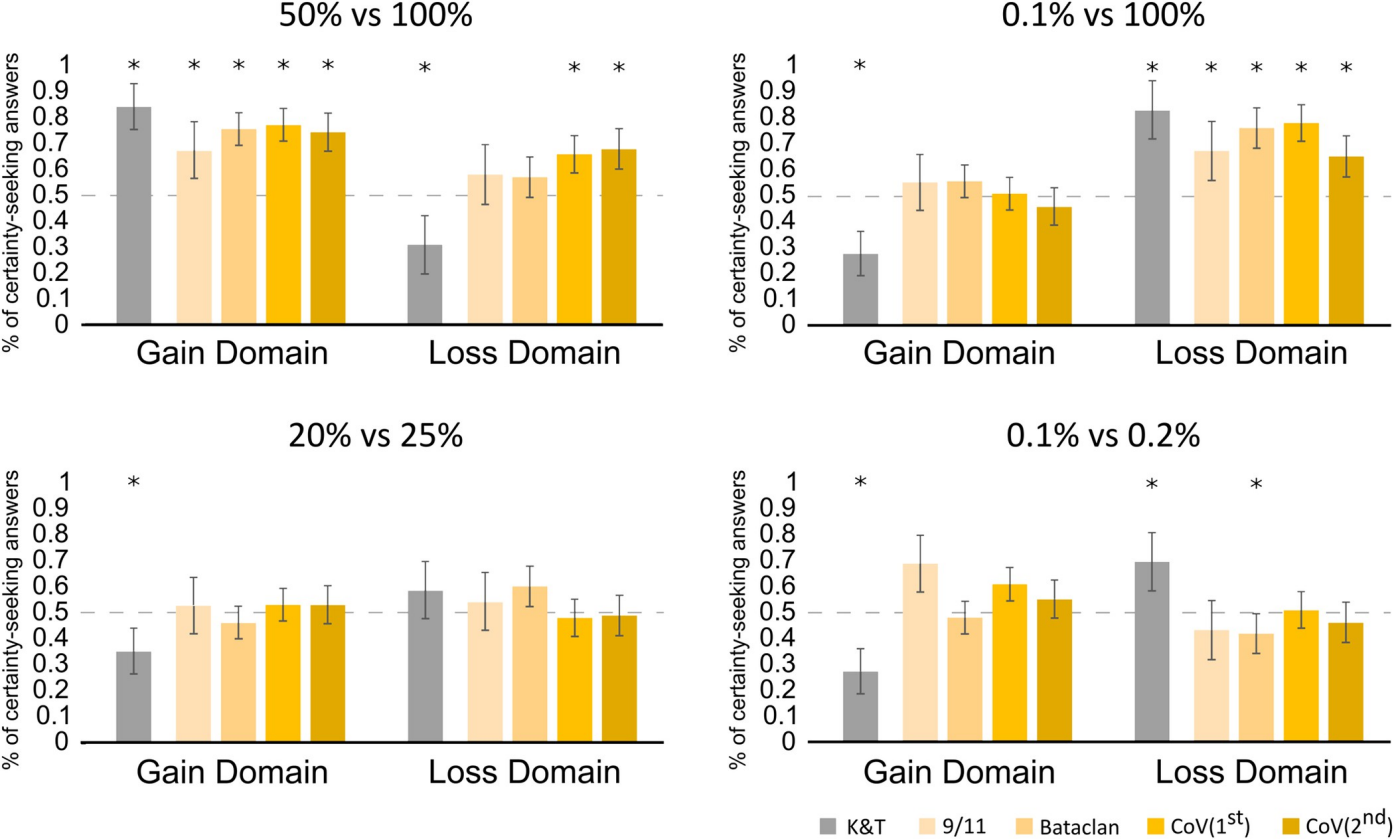
Percentage of individuals choosing the most likely option, by experiment: K&T (Kahneman and Tversky, 1979) [5]; 9/11 (Sacco, Blanzieri and Galetto, 2003) [9]; Bataclan (Experiment 1 of the present paper); Cov 1st (Experiment 2 of the present paper, lockdown period); Cov 2nd (Experiment 2, re-openings period). The four panels represent the four couples of prospects included in this study. Each bar represents the proportion that the prospect with a higher outcome probability was selected (e.g., 25% in 20% vs. 25% option). Error bars represent 2 standard errors; asterisks = p < 0.05. Chi squared tests for Kahneman and Tversky (1979) computed on the basis of the published data.

The response distribution differed substantially from chance for items A (χ^2^ = 52.36, p_c_<0.001), A’ (χ^2^ = 19.11, p_c_<0.001), B’ (χ^2^ = 54.56, p_c_<0.001), but not for B (χ^2^ = 0.09), C (χ^2^ = 0.56), C’ (χ^2^ = 0.36) D (χ^2^ = 3.27) or D’ (χ^2^ = .02; all corrected p values non significant). There was no association between gender and the answers to any prospect (all p_c_ > 0.05). 77% of the respondents chose the option with the highest probability of happening in prospect A, 66% e in prospect A’, 51% in prospect B, 78% in prospect B’, 53% in prospect C, 48% in prospect C’, 57% in prospect D and 51% in prospect D’. Fig 1 shows the relative frequencies of the more likely options (e.g.: percentage of people choosing a sure win of 2000 € over 50% chance of winning 4000 €) for each couple of prospects in Experiment 1, as well as for the data gathered during the pandemic (Experiment 2) and reference values drawn from previous work [5,9].

#### Response distributions (2^nd^ time period–summer 2020)

The response distribution differed substantially from chance for items A (χ^2^ = 31.1, p_c_<0.001), A’ (χ^2^ = 17.85, p_c_<0.001), B’ (χ^2^ = 15.11, p_c_<0.001), but not for B (χ^2^ = 1.03), C (χ^2^ = 0.46), C’ (χ^2^ = 0.03), D (χ^2^ = 1.8) or D’ (χ^2^ = 1.03, all corrected p values non-significant). An association was found between gender and the answer to prospect D’ (χ^2^ = 4.46), but it was found to be non-significant after correction for multiple comparisons. 74% of the respondents choose the option with the highest probability of happening in prospect A, 68% in prospect A’, 46% in prospect B, 66% in prospect B’, 53% in prospect C, 49% in prospect C’, 56% in prospect D and 46% in prospect D’.

#### Correlations with personality traits and epidemiological data

The answers to the questions were not significantly correlated with levels of worry for the pandemic, self-perceived information about the pandemic or metrics related to the Covid-19 pandemic (all FDR-corrected p > 0.05). It is, however, worth noting that only 3.8% reported to be not informed or not very informed about the pandemic; 13% not preoccupied or slightly preoccupied about the health consequences and 7% not preoccupied or slightly preoccupied about the economic consequences. Only two correlations between personality and answers to the questions were significant after FDR correction: stability and A (r = -.19, pFDR = .017) and consciousness and A’ (r = -.19, pFDR = .035).

#### Robustness checks

While pooling all our three experiments, our results differed from the Italian subsample of Ruggeri and colleagues, for the majority of prospects: A (χ^2^ = 34.11, p_c_<0.001), A’(χ^2^ = 6.66, p_c_ = 0.03), B(χ^2^ = 145.52, p_c_<0.001), C(χ^2^ = 11.89, p_c_ = 0.002), D (χ^2^ = 102.89, p_c_<0.01) and D’ (χ^2^ = 15.19, p_c_<0.001), but not for prospects B’ (χ^2^ = 0.10) or C’(χ^2^ = 2.88). In prospects A, A’, B and D we observed a higher proportion of more certain prospects with respect to Ruggeri and colleagues (76% vs 62% for A; 64% vs 58% for A’, 57% vs 31% for B), while in C and D’ we did not notice a distribution different from chance, in line with what was noted in the analysis of the single experiments.

Expanding the reference sample (Ruggeri et al.) to include all the countries, while keeping the same demographical profile, yielded slightly different results, as the prospects A’(χ^2^ = 87.36, p_c_<0.001), B(χ^2^ = 68.15, p_c_<0.001), B’(χ^2^ = 7.88, p_c_ = 0.03), D (χ^2^ = 95.73, p_c_<0.001) and D’ (χ^2^ = 26.68, p_c_<0.001) resulted different from the replication, while A(χ^2^ = 1.14), C(χ^2^ = 1.07) and C’(χ^2^ = 0.65) did not. As in the previous case, we observe a larger proportion of more certain prospects for choices A’ (64% vs 42% for Ruggeri et al), B (57% vs 38%) and B’ (68% vs 61%), and, at odds with Ruggeri and colleagues a distribution of preference not different from chance level for prospect D’.

Lastly, comparing our results with Sacco et al. finds a significant difference for prospects A (χ^2^ = 16.05, p_c_<0.001), A’ (χ^2^ = 7.15, p_c_ = 0.038), B (χ^2^ = 12.64, p_c_ = 0.002), and D (χ^2^ = 47.94, p_c_<0.001), but not for B’(χ^2^ = 0.1), C(χ^2^ = 0.22), C’(χ^2^ = 0.9), and D’(χ^2^ = 2.32), and observed for choices A and A’ a larger proportion (see Fig 1) of more certain prospects with respect to Sacco et al [9].

## Discussion

Prospect Theory [5] demonstrated that people do not make rational decisions based on expected utility, but are instead biased by specific cognitive tendencies leading to neglect, under- or over- consider information, depending on the context of presentation. Ruggeri and colleagues [8] recently replicated the theory’s main findings, despite some divergences.

A previous paper [9] started to investigate changes in decision-making in the presence of globally challenging events, suggesting a tendency towards risk aversion after the terrorist attack of 9/11. However, subsequent papers found contrasting results. On one side, Li and colleagues [11] showed that, after a natural disaster, people tend to overweight small probabilities favouring both protection from rare but catastrophic events and very small chances to reach a large gain. Voors and colleagues [22] found more risk-seeking behavior after a violent conflict in Burundi, as well as altruistic behaviour. Eckel and colleagues and Shupp and colleagues [23,24] found mixed results after two different hurricanes, and Page and colleagues [25] found that homeowner victims of the 2011 floods were more risk-seeking, as they were more likely to choose risky gambles. On the other side, and in agreement with Sacco and colleagues, other studies found that an external shock, such as a natural catastrophe, can increase risk aversion (e.g., Cameron and Shah [26] after floods or earthquakes in rural Indonesia; Cassar and colleagues [27] after the 2004 tsunami in Thailand; Reynaud and Aubert, [28] after floods in Vietnam, and Beine and collaborators [29] after earthquakes in Albania). The picture is therefore still unclear. Here, we present data gathered after different international events generating global disquiet: the first event (2015 Paris terror attack) was very similar to that studied by Sacco et al. [9] as it was again a terrorist attack, while the second one, the Covid-19 pandemic, was very different in nature.

We discuss our results (both Experiment 1 and Experiment 2) in light of previous data [9] and of the original Kahneman and Tversky results ([5], K&T henceforth).

In Prospect A, all studies showed a significant preference for the sure win of medium entity with respect to a 50% probability of winning double the sum. Very differently, in Prospect A’, the preference for a probable loss of greater magnitude, found by K&T, was not replicated in either Sacco and colleagues or in the experiments of the present study. During the Covid pandemic the choice for the safest option is significantly preferred, showing a heightened loss aversion and an inversion of preferences with respect to the PT previsions. In Prospect B the overweighting occurring when subjects had to choose between a small sure win or a very unlikely larger win (0.1% of winning, equivalent to the choice made when considering to buy a lottery ticket), found by K&T, was not replicated in either Sacco and colleagues [9] or in the experiments of the present study. On the contrary, in Prospect B’, the overweighting of very low probabilities to suffer a loss was significantly present in all studies. We found fewer striking results for Prospects C-C’ and D-D’. Indeed, in Prospect C-C’, which involved similar alternatives, the preference for the slightly lower probability of winning a bigger amount of money disappeared during all periods of uncertainty. On the other hand, a weak (not significant) tendency to choose the slightly higher probability of losing a smaller amount of money was present in all the studies reported in Fig 1. In Prospect D-D’, concerning very unlikely events, both the preference for the less probable event of winning a bigger amount of money and the more probable event of losing a smaller amount of money, found by K&T, were not replicated in either Sacco and colleagues or in the experiments of the present study.

It is possible that sociodemographic characteristics of our sample, such as age [30] and nationality can, in part, explain why our findings diverged from Tversky and Kahnemann’s. For instance, [31] estimated the parameters used in the formulation of the Cumulative Prospect Theory in 53 nations, finding substantial heterogeneity. However, it is worth noting that the authors report that 94% of the Italian participants shows the reflection effect, which is largely lost in the data presented here (prospects B-B’). Furthermore, drawing age-matched subsamples from the recent replication effort by Ruggeri and collaborators does not significantly change the scenery: even when comparing our results with more recent and age-matched reference samples drawn during times not characterized by natural disasters or other disrupting events, our data show a heightened preference for less uncertain prospects. Furthermore, the results of our experiments largely overlapped those obtained by Sacco et al. [9], showing an even greater preference for less uncertain options in prospects A and A’. More in general, and taking in consideration their entire sample, Ruggeri and colleagues completely replicated Prospects A-A’ and B-B’, i.e., those comparing probable vs. sure prospects. On the contrary, all our data–collected during different very uncertain historical periods–show a preference towards outcomes marked by no uncertainty. The other prospects, those where options with different probabilities are compared, were not completely replicated by Ruggeri and colleagues. In particular, in Prospect C Ruggeri could not replicate the effect originally found by K&T in the gain domain, and in Prospect D’ Ruggeri found a strong attenuation of the effect found by K&T in the loss domain. These results are in line with what we found in all our experiments. Therefore, while the tendencies characterizing Prospects A-A’ and B-B’ seem to be specific of periods of uncertainty, the same conclusion cannot be stated for decisions taken in Prospects C-C’ and D-D’. In particular, prospects C was not replicated by Ruggeri and colleagues and in C’ even Kahneman and Tversky [5] found no preference between the options. Besides, distributions of answers in C-C’ and D-D’ are, in large part, not different from chance, showing indifference between the options. It must be said that the two options presented in these two pairs of prospects were similar in the probability of gain/loss and that the probabilities of winning/losing could be very small (D-D’): choices made between options that seem very similar could therefore be less salient.

A few other studies analyzed risk preferences during the Covid-19 pandemic. Most of them come from the economic literature and thus the methodologies used differ from that of the present work. Therefore, their results cannot be directly compared with ours, but they nonetheless give interesting suggestions. Some studies failed to detect any change in risk preferences before and after the start of the pandemic [32–34]. Angrisani and colleagues, for instance, use a laboratory task (Bomb Risk Elicitation Task) to test risk preference in a group of subjects before (February-March 2019) and after (April 2020) the emergence of COVID-19, finding no shift in risk preferences. A similar stability of risk preferences was noted by Drichoutis and Nayga and Lohmann, whose studies included a task similar to the one used in this study (Lottery choice) and used a student sample, like we did. While our results are largely at odds with Angrisani and colleagues, we nevertheless agree on their conclusion that the risk preferences in the experimental task are not modified by negative expectations on future financial situation. Different results come from longitudinal study that tried to assess Prospect Theory parameters [35] during the early stages of the COVID-19 pandemic (March 13 to May 11, 2020), and found an increased tolerance of non-tail risks, but a decreased tolerance of tail (extreme) risks, in the loss domain. In partial agreement with them, in this study we found a stronger search for security for the prospects (B-B’) characterized by tail risks, both in the loss and gain domain, but observed the same behavior in prospects (A-A’) characterized by non-tail risks. Shachat et al. [36], using similar tasks, actually found a more complex picture: beside an increase in prosocial and cooperative behavior and an increased risk tolerance in the gain domain during the early stages of the pandemic, they found a decrease in risk tolerance in the loss domain. Besides, they found a transient correlation between the decrease of risk and ambiguity tolerance and the death of a prominent doctor, Li Wenliang, involved in the early stages of the fight against Covid-19. Their results in the loss domain are in line with the main result of the present paper, namely the cognitive tendency to risk aversion during periods of crisis. In the same direction, Bu and colleagues [37] found that exposure to the virus leads to an increase in risk aversion, and that as the exposure increases (i.e., residents of the city of Wuhan vs. residents of Hubei vs. residents of other provinces of China), risk aversion increases too, as measured both by lower amounts of planned risk taking and by lower allocation of money to risky investment decisions.

Therefore, this study is not alone in identifying increased risk avoidance, and this finding could be explained by different mechanisms. For instance, Ikeda and collaborators [35] suggested that stress could be the driver behind their results. Their hypothesis stems from earlier results [38] in which experimenters manipulated stress hormone levels by administering doses of hydrocortisone, and found that this increased risk aversion and overweighting of small probabilities in the gain domain.

A more recent study [39] supports the idea, as it found that individual with chronic anxiety disorders exhibited enhanced levels of risk aversion relatively to healthy controls, but this did not extend to of loss aversion. A growing literature is highlighting the link between anxiety and negative disposition towards risk and uncertainty [40].

Psychological stress is then a strong candidate to explain our results: participants in the present study reported both a high level of information and high levels of worry related to the pandemic and its consequences. This conclusion is reinforced by the fact that, as it could be expected, early studies reported psychological distress linked to the COVID-19 pandemic [41].

The role of stress could be mediated by limbic mechanisms specific to shocking public events, especially for the ones that can cause “flashbulb memories” [42].

However, Sharot et al. [43] found evidence of such limbic mechanism only for people that directly experienced traumatic events (such as being in the immediate proximity of the World Trade Center)–a condition that, in this study, could only hold for the Covid 19 experiment. Interestingly, the idea that psychological stress is a possible explanation for our results is also in line with the results of [44], who found increased risk aversion after elicitation of fearful emotions (namely, watching short fragments of horror movies).

Other causes, such as the income effect [25], caused by economic damages and loss of earning opportunities, have been linked to risk aversion. Both the composition of our samples and the fact that we observed a similarly heightened risk aversion after events characterized by wildly varying economic effects (doubtlessly lower for the Bataclan attacks with respect to the 9/11 terror attacks or the COVID-19 pandemic) push us away from supporting explanations simply based on economic effects. However, the lack of socioeconomical data makes it hard to either prove or disprove this specific hypothesis.

Furthermore, this study did not have the aim to support or disconfirm a specific decision making model: while we note that some authors [45,46] found, in human and primates, evidence of flexible decision making strategies (i.e.: shifting from multiplicative to additive combination of reward magnitude and probability), dependent on the task circumstances and the sequence of the events (i.e.: pseudorandom sequence of choices versus blocks of repeated choices), this study did not contrast different decision-making scenarios, nor was the limited set of prospects used in this replication study sufficient to estimate prospect theory parameters, as even the full set of prospects replicated by Ruggeri et al is “[…] grossly insufficient when it comes obtaining precise and reliable estimates of Prospect Theory parameters” [47] and further studies are needed, both to explore in more detail what could be the main driver behind the phenomenon described in this study and to test different models of decision making after a natural catastrophe.

In summary, this study replicates the findings of Sacco and colleagues [9], showing an increase in loss aversion after an external shock. The effect is not substantially modulated by personality factors, nor by the type of event (it generalizes across very different situations, from Covid pandemic to terrorist attacks), nor by its instantaneous perceived magnitude (1st versus 2nd wave of data gathered during Covid-19), levels of fear, or damage caused by the event (measured as loss of lives, daily admissions to ICUs and daily variation in the number of deaths): it seems that such variables have little to no effect when there is a sudden and substantial increase in the level of background risk.
